# Hydroxyl Group Acetylation of Quercetin Enhances Intracellular Absorption and Persistence to Upregulate Anticancer Activity in HepG2 Cells

**DOI:** 10.3390/ijms242316652

**Published:** 2023-11-23

**Authors:** Kozue Sakao, Hanako Saruwatari, Shohei Minami, De-Xing Hou

**Affiliations:** 1 The United Graduate School of Agricultural Sciences, Kagoshima University, Kagoshima 890-0065, Japan; sakaok24@agri.kagoshima-u.ac.jp; 2 Department of Food Science and Biotechnology, Faculty of Agriculture, Kagoshima University, Kagoshima 890-0065, Japan; k2372356@kadai.jp (H.S.); k5157734@kadai.jp (S.M.)

**Keywords:** quercetin, acylation, bioavailability, apoptosis, metabolites

## Abstract

Quercetin, a flavonoid compound widely distributed in many plants, is known to have potent antitumor effects on several cancer cells. Our previous study revealed that the acetylation of quercetin enhanced its antitumor effect. However, the mechanisms remain unknown. This study aimed to elucidate the bioavailability of acylated quercetin in the HepG2 cell model based on its antitumor effect. The positions of quercetin 3,7,3′,4′-OH were acetylated as 3,7,3′,4′-*O*-tetraacetylquercetin (4Ac-Q). The inhibitory effect of 4Ac-Q on HepG2 cell proliferation was assessed by measuring cell viability. The apoptosis was characterized by apoptotic proteins and mitochondrial membrane potential shifts, as well as mitochondrial reactive oxygen species (ROS) levels. The bioavailability of 4Ac-Q was analyzed by measuring the uptake and metabolites in HepG2 cells with high performance liquid chromatography (HPLC)—photodiode array detector (PDA) and—ultraviolet/visible detector (UV/Vis). The results revealed that 4Ac-Q enhanced the inhibitory effect on HepG2 cell proliferation and induced its apoptosis significantly higher than quercetin. Protein array analysis of apoptosis-related protein indicated that 4Ac-Q increased the activation or expression of pro-apoptotic proteins, including caspase-3, -9, as well as second mitochondria-derived activator of caspases (SMAC), and suppressed the expression of apoptosis inhibiting proteins such as cellular inhibitor of apoptosis (cIAP)-1, -2, Livin, Survivin, and X-linked inhibitor of apoptosis (XIAP). Furthermore, 4Ac-Q stimulated mitochondrial cytochrome *c* release into the cytosol by enhancing ROS level and depolarizing the mitochondrial membrane. Finally, the analysis of uptake and metabolites of 4Ac-Q in HpG2 cells with HPLC-PDA and -UV/Vis revealed that 4Ac-Q was metabolized to quercetin and several different acetylated quercetins which caused 2.5-fold higher quercetin present in HepG2 cells than parent quercetin. These data demonstrated that acetylation of the quercetin hydroxyl group significantly increased its intracellular absorption. Taken together, our findings provide the first evidence that acetyl modification of quercetin not only substantially augments the intracellular absorption of quercetin but also bolsters its metabolic stability to elongate its intracellular persistence. Therefore, acetylation could serve as a strategic approach to enhance the ability of quercetin and analogous flavonoids to suppress cancer cell proliferation.

## 1. Introduction

Quercetin, known as a flavonol-type flavonoid, is a widespread polyphenol found in many vegetables, fruits, and tea. The pharmacological effects of quercetin, including antioxidant, anti-inflammatory, anti-atherosclerotic, anti-tumor, antihypertensive, and prevention of cerebrovascular diseases, have been reported [1]. Consuming large amounts of quercetin is considered to decrease the risk of lung cancer [2], stomach cancer [3], and colon cancer [4,5]. Partial data indicated that quercetin could inhibit hepatocellular carcinoma cell growth by inducing apoptosis/cell death and cell cycle arrest. The combination of cancer therapy of fluorouracil (5-FU) with quercetin showed an additive or synergistic inhibitory effect on hepatocellular carcinoma cell growth [6]. Quercetin could also enhance the anticancer effects of doxorubicin chemotherapy on hepatocellular carcinoma cells, companying with the protection of normal hepatocytes [7].

Therefore, quercetin is considered a promising chemopreventive compound for potential chronic disease-preventive effects. On the other hand, quercetin has low bioavailability due to poor absorption [8,9] and rapid metabolism. Those properties limit the potential application of quercetin. Recent studies have attempted to improve its bioavailability and bioactivity by modifying quercetin structure with various chemical methods [10,11,12]. Acylation is one such modification, especially the *O*-acylation of quercetin, which has been reported to improve various biological activities, such as anticancer, antivirus, antiplatelet, and cytoprotection effects [13,14,15,16]. In our previous studies, we replaced the hydroxy groups of quercetin with methyl, benzyl, and acetyl groups to evaluate the changes in their bioactivity [17,18]. Of which, 3,7,3′,4′-*O*-tetraacetylquercetin (4Ac-Q), which was acylated at the 3,7,3′,4′-OH positions of quercetin revealed a higher ability of apoptosis induction in myeloid human leukemia cancer cells (HL-60 cell). However, the molecular regulators of the higher apoptotic response are not fully understood, and the effects of 4Ac-Q on intracellular absorption, metabolism, and bioavailability remain unknown. This study aimed to elucidate the bioavailability of 4Ac-Q by the HepG2 cell model based on its antitumor effect. The inhibitory effect of 4Ac-Q on HepG2 cell proliferation was assessed by measuring cell viability. The apoptosis was characterized by proteomic analysis of 35 human apoptosis-related proteins and mitochondrial membrane potential shifts, as well as mitochondrial reactive oxygen species (ROS) levels. To finally obtain an understanding of how quercetin bioavailability is altered by acetylation, the cellular uptake and metabolism of 4Ac-Q and parent quercetin in HepG2 cells were evaluated by measuring their metabolites with HPLC-PDA and -UV/Vis. Our study will clarify the effects of the *O*-acylation of the quercetin hydroxyl group on its bioavailability and antitumor activity.

## 2. Results

### 2.1. 4Ac-Q Enhances the Inhibitory Effect on HepG2 Cell Proliferation

**Figure 1:**
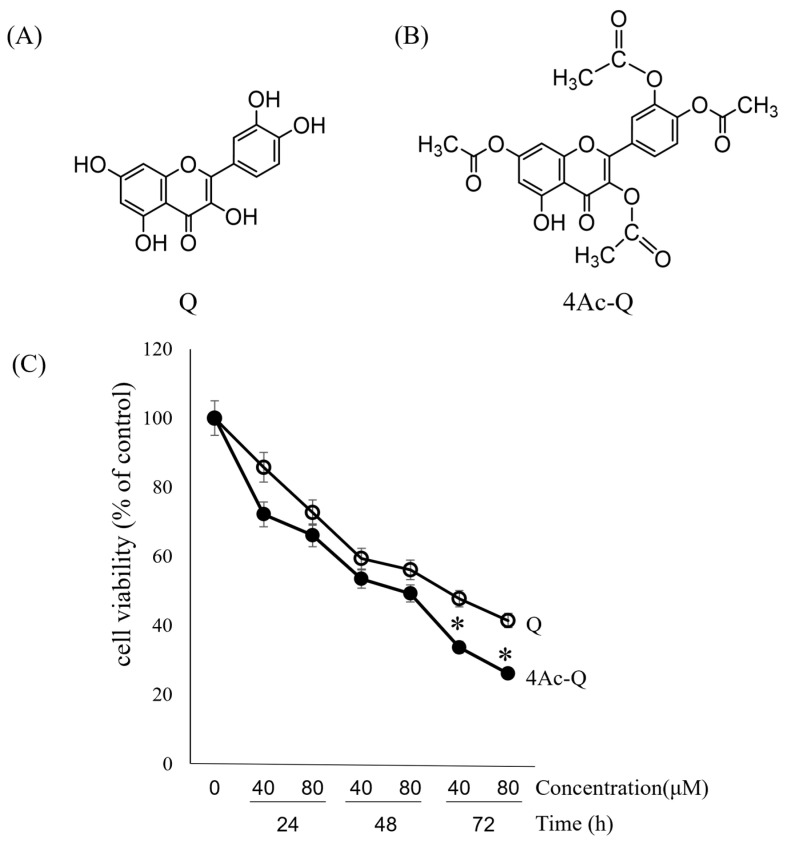
Chemical structure of quercetin (Q) (**A**) and 4Ac-Q (**B**), and the viability of HepG2 cells treated with Q or 4Ac-Q in the indicated times and doses (**C**). Cells were treated with 40 μM and 80 μM of Q or 4Ac-Q for 24, 48, and 72 h, respectively. Cell viability was determined via MTT assay. Data from at least three independent triplicated experiments were presented as mean ± SD, n = 9, and * mark denoted significant differences (* *p* < 0.05) between Q and 4Ac-Q.

In a previous study, the cell growth inhibitory effects of quercetin on HepG2 cells revealed a concentration- and time-dependent manner in an 3-(4,5-dimethyl-2-thiazolyl)-2,5-diphenyltetrazolium bromide (MTT) assay. A significant inhibition of cell growth was observed after 48 h treatment with an IC_50_ value of 76.1 μM [19]. Therefore, in this study, to compare the inhibitory effect of quercetin and 4Ac-Q on HepG2 cell proliferation, both dose- and time-course dependence were investigated via MTT assay. As shown in Figure 1C, 4Ac-Q significantly enhanced the inhibitory effect on HepG2 cell proliferation at 72 h in both 40 μM and 80 μM than quercetin, although both quercetin and 4Ac-Q inhibited HepG2 cell proliferation in a dose- and time-dependent manner.

### 2.2. 4Ac-Q Enhances Cell Death via Both Apoptosis and Necrosis

**Figure 2:**
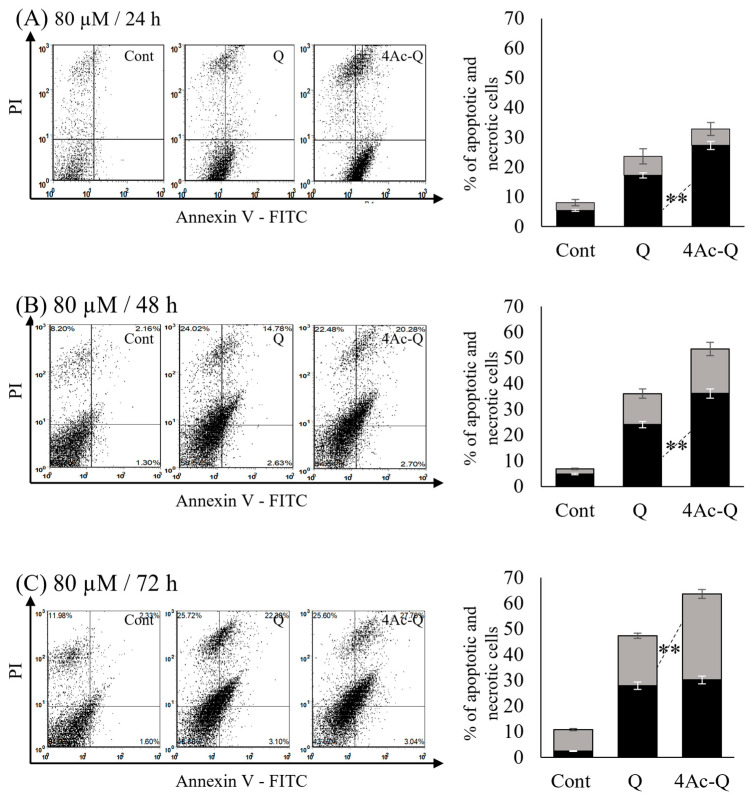
4Ac-Q and quercetin (Q)-induced apoptosis and necrosis in HepG2 cells. HepG2 cells were treated with 80 µM 4Ac-Q or Q for (**A**) 24 h, (**B**) 48 h, and (**C**) 72 h, respectively. Left panels indicated the flow cytometry pattern of Annexin V-FITC/PI fluorescence intensity, and right panels showed the quantitative graph of apoptotic (black column) and necrotic (gray column) cell fractions. Results are expressed as the percentage of cell death of HepG2 cells treated with 0.1% dimethyl sulfoxide (DMSO) (Cont) or 4Ac-Q or Q (mean ± SD, n = 3) and statistically analyzed using one-way ANOVA followed by Tukey’s test. Data from at least three independent triplicated experiments are presented as mean ± SD, n = 9, and * mark denoted significant differences (** *p* < 0.01) between Q and 4Ac-Q.

Next, we evaluated whether the proliferation inhibition of HepG2 cells by 4Ac-Q was due to apoptosis induction. After staining cells with Annexin V- fluorescein isothiocyanate (FITC) and propidium iodide (PI), 25,000 cells were counted by flow cytometry and sorted into apoptotic fraction (early (FITC+/PI−) + late (FITC+/PI+) and necrotic fraction (FITC−/PI+). Cell death was induced for 24–72 h with 80 μM of Q or 4Ac-Q. As shown in Figure 2, 4Ac-Q induced significantly higher apoptosis than quercetin at 24 h (Figure 2A) and 48 h (Figure 2B). Interestingly, 4Ac-Q induced significantly higher necrosis than quercetin at 72 h with a similar apoptosis as quercetin (Figure 2C). Consistent with the cell viability data (Figure 1C), 4Ac-Q enhanced inhibitory effectiveness on cell proliferation inhibition via increasing both apoptosis and necrosis at 24–48 h, and mainly via increasing necrosis after 72 h, compared with parent quercetin.

### 2.3. 4Ac-Q Significantly Regulates the Level of Apoptosis-Related Proteins Than Quercetin

**Figure 3:**
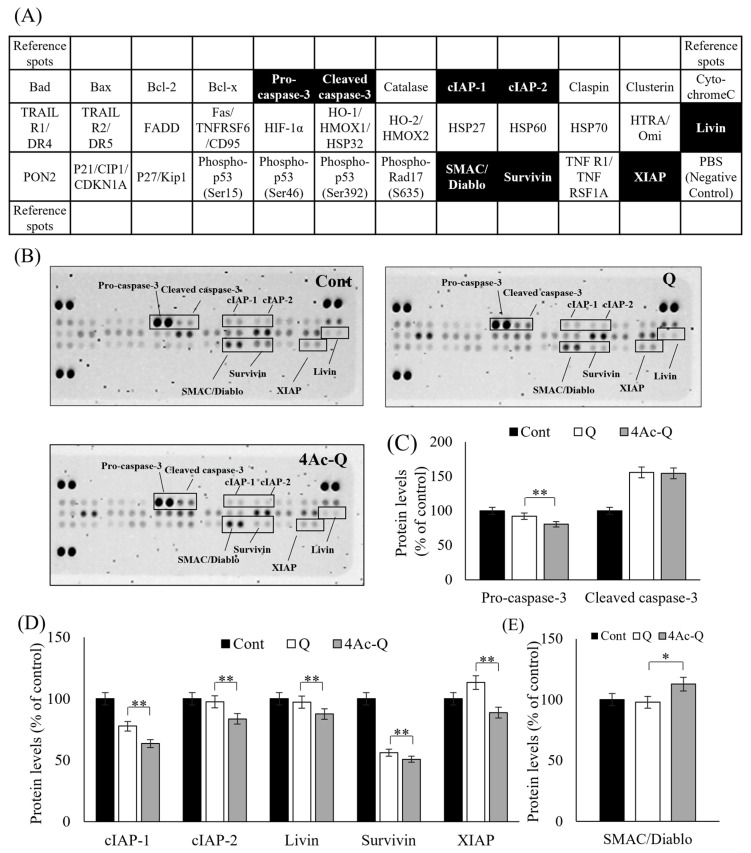
Protein array analysis of 35 apoptosis-related proteins in HepG2 cells treated with quercetin (Q) and 4Ac-Q. (**A**) The location of each apoptosis-related protein on the human apoptosis antibody array membrane. Of which, the protein with significant change was labeled in the black boxes. (**B**) Representative array images indicating the protein levels of various apoptosis-related proteins in HepG2 cells treated with 0.1% DMSO (Cont), 80 μM Q, or 4Ac-Q for 24 h. (**C**) The mean pixel density of pro-caspase-3 and cleaved caspase-3 in the Q or 4Ac-Q-treated group relative to the non-treated group (Cont). (**D**) The mean pixel density of IAP family proteins including cIAP-1 and -2, Livin, Survivin, and XIAP. (**E**) The mean pixel density of IAP antagonist SMAC/Diablo. * *p* < 0.05, ** *p* < 0.01, significant differences between quercetin and 4Ac-Q.

To fully understand the mechanisms of 4Ac-Q-induced cell death, we first used protein array analysis of 35 apoptosis-related proteins (Figure 3A) to compare the expression level of each protein between Q and 4Ac-Q treatment (Figure 3B). As shown in Figure 3C, cleavage of caspase-3 and reduction in procaspase-3 were significantly detected in the 24 h treatment with 80 μM of Q and 4Ac-Q (Figure 3C). On the other hand, 4Ac-Q significantly suppressed the expression of apoptosis inhibitor proteins, including cellular inhibitor of apoptosis (cIAP-1), -2, Livin, Survivin, and X-linked inhibitor of apoptosis (XIAP) (Figure 3D). Furthermore, the expression level of the mitochondrial pro-apoptotic protein second mitochondria-derived activator of caspase/direct inhibitor of apoptosis-binding protein with low pI (SMAC/Diablo), which is involved in the execution of apoptosis by inhibiting the IAP family [20], was found to be significantly higher in 4Ac-Q treatment than that in quercetin treatment (Figure 3E). Meanwhile, there was no change in the expression of high temperature requirement A2 (HtrA2)/Omi, which inhibits the function of IAP, as well as SMAC/Diablo.

**Figure 4:**
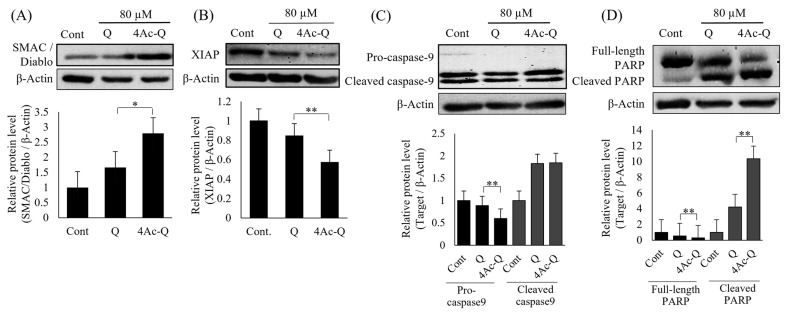
The typic expressions of some apoptosis factors were confirmed by Western blotting. HepG2 cells were treated with 80 µM of quercetin (Q) or 4Ac-Q for 24 h or with 0.1% DMSO (Cont). Protein levels of SMAC/Diablo (**A**), XIAP (**B**), Caspase-9 (**C**), and PARP (**D**) in HepG2 cells were detected by Western blotting. Quantification data were normalized by β-Actin level, and each value represents mean ± SD of 3 repeated experiments. * *p* < 0.05, ** *p* < 0.01, significant differences between quercetin and 4Ac-Q.

Furthermore, to check the accuracy of protein array analysis results, some important factors in the regulation of apoptosis, such as SMAC/Diablo, procaspase-9, XIAP, and Poly (ADP-ribose) polymerase (PARP), were reconfirmed via Western blotting (Figure 4). Almost all of them showed a similar expression pattern as protein array analysis data. In comparison with quercetin, 4Ac-Q significantly increased the protein expression of SMAC/Diablo (Figure 4A) and significantly reduced the protein expression of XIAP (Figure 4B), procaspase-9 (Figure 4C), as well as full PARP (Figure 4D).

### 2.4. Mitochondrial Dysfunctions Are Involved in 4Ac-Induced Apoptosis

**Figure 5:**
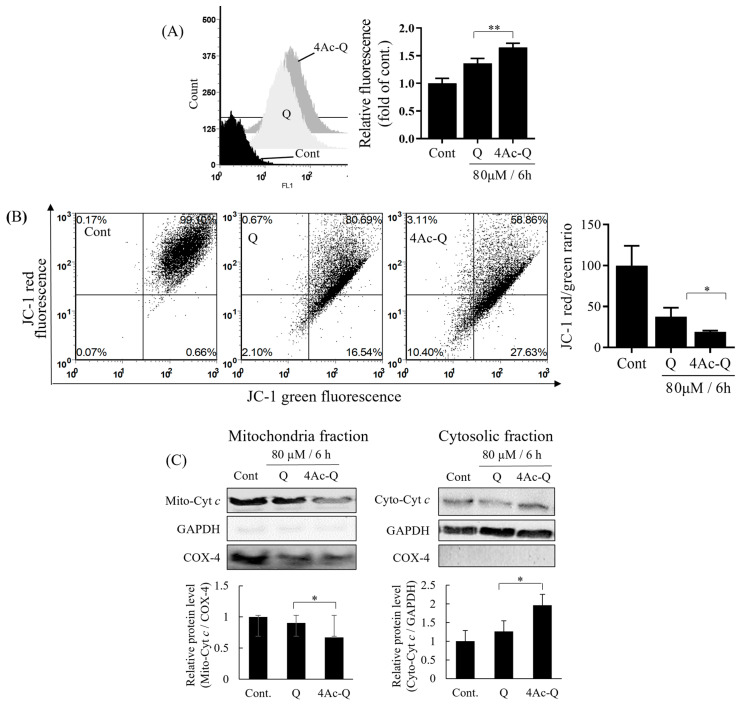
Mitochondrial dysfunctions by quercetin (Q) and 4Ac-Q. (**A**) Mitochondrial ROS generation was monitored by MitoSOX Red mitochondrial superoxide chemical probe. ROS level was expressed as relative fold to control HepG2 cells (Cont) treated with 0.1%DMSO. (**B**) Alterations in mitochondrial membrane potential (Ψm) were measured by flow cytometry using the JC-1 staining method, and the ratio of red/green fluorescence intensity was presented asΨm). (**C**) Cytochrome *c* release. Mitochondrial (Mito-Cyt *c*) and cytosolic fractions (Cyto-Cyt *c*) were fractionated, and cytochrome *c* release was detected by Western blotting. The amount of cytochrome *c* in the cytosolic or mitochondrial fractions was normalized, respectively, by compartment-specific glyceraldehyde-3-phosphate dehydrogenase (GAPDH) or mitochondrial cytochrome c oxidase subunit 4 (COX-4) protein levels. Data from at least three independent triplicated experiments are presented as mean ± SD, and * mark denoted significant differences (* *p* < 0.05, ** *p* < 0.01, between quercetin and 4Ac-Q).

Mitochondria have been reported to play a key role in the regulation of apoptosis [21]. Especially, extra ROS production triggers mitochondrial dysfunctions, including the loss of mitochondrial membrane potential and the release of cytochrome c from the mitochondrion. Thus, we first measured the ROS levels with MitoSOX Red, a chemical probe that selectively reacts with superoxide in the mitochondrion. As shown in Figure 5A, 4Ac-Q produced significantly higher ROS level than quercetin, although both quercetin and 4Ac-Q caused a significant increase in mitochondrial ROS levels at 6 h compared to control. Next, we measured membrane potential loss by JC-1 staining, a membrane-permeable fluorescent probe that can measure mitochondrial membrane potential. As shown in Figure 5B, 4Ac-Q decreased significantly mitochondrial membrane potential more than quercetin, although both quercetin and 4Ac-Q caused a significant decrease in mitochondrial membrane potential at 6 h compared to control. Furthermore, we separated cytoplasmic and mitochondrial fractions from HepG2 cells treated with quercetin (Q) or 4Ac-Q or 0.1% DMSO (Cont) and then detected the protein levels by Western blotting (5A). Both quercetin and 4Ac-Q significantly increased cytoplasmic cytochrome *c* levels and significantly reduced mitochondrial cytochrome *c* levels compared to control. 4Ac-Q showed significantly stronger actions than quercetin. These data demonstrated that 4Ac-Q induced apoptosis by ROS-mediated mitochondrial dysfunction pathway.

### 2.5. 4Ac-Q Enhances the Cellular Uptake in HepG2 Cells

The above data indicated that 4Ac-Q induced HepG2 cell apoptosis in a similar apoptotic pathway as quercetin, but with stronger ability. It caused us to speculate that the acetylation of the hydroxyl group of quercetin may affect the absorption and/or metabolism of quercetin to improve its bioactivity. To clarify this speculation, we first determined the amounts of quercetin and 4Ac-Q taken up in HepG2 cells by HPLC-PDA and -UV/Vis detector with selectable wavelengths ranging from UV to visible light.

**Figure 6:**
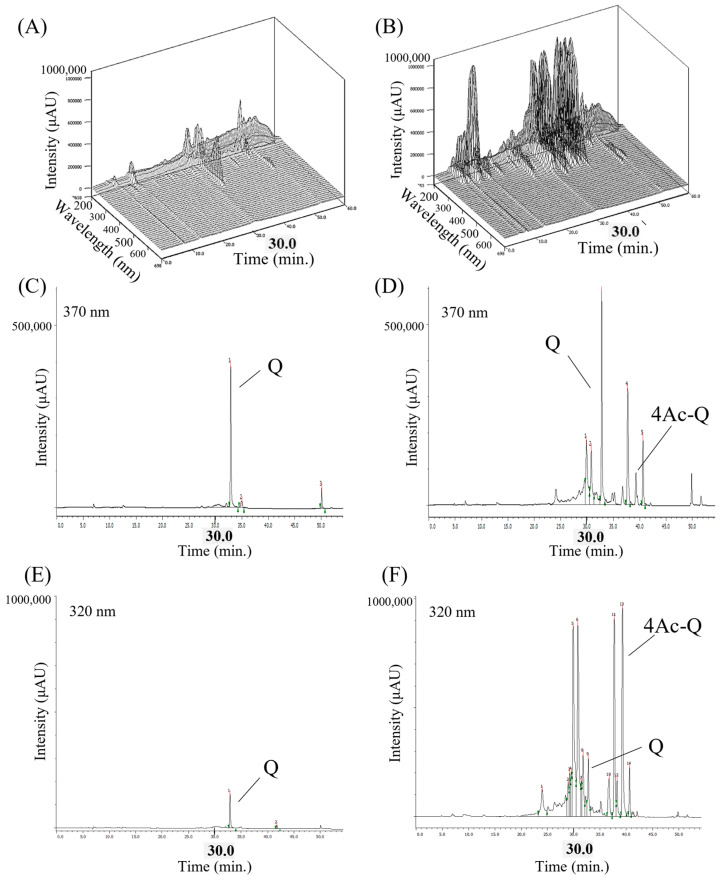
Chemical profile of HepG2 intracellular extract analyzed by HPLC-PDA and -UV/VIS. (**A**,**B**) Three-dimensional chromatograms of intracellular extracts from quercetin or 4Ac-Q-added HepG2 cells for 3 h detected by HPLC-PDA (wavelength: 210–700 nm). Three-dimensional HPLC was represented by a 3D chromatogram with the retention time, absorption wavelength, and peak intensity of the components as the three axes. (**C**,**D**) HPLC-UV/Vis chromatograms of quercetin at maximum absorption wavelength (370 nm). (**E**,**F**) HPLC-UV/Vis chromatograms of 4Ac-Q at maximum absorption wavelength (320 nm).

Figure 6A shows a three-dimensional chromatogram of quercetin-added HepG2 cells via HPLC-PDA detection. One major peak (retention time 32.82 min) and two smaller peaks were observed. The retention time of the major peak was consistent with the dominant peak in the chromatogram shown in Figure 6C, which was monitored at 370 nm, a characteristic wavelength of quercetin, and was assumed to be quercetin. On the other hand, the three-dimensional chromatogram of 4Ac-Q-added HepG2 cells showed several peaks. Of which, the retention time of the major peak was consistent with the dominant peak in the chromatogram shown by Figure 6F, which was monitored at 320 nm, a characteristic wavelength of 4Ac-Q, and was assumed to be 4Ac-Q. Besides this, the peak at a retention time of 32.74 min was consistent with that of quercetin, shown in Figure 6D. Other peaks are unknown metabolites. In comparison of quercetin levels obtained from Figure 6C,D, approximately 2.5-fold higher quercetin was observed in 4Ac-Q-added HepG2 cells than by quercetin addition. It is worth noting that the total peak area of the chromatograms in the 4Ac-Q treatment was quite larger than that of quercetin. This suggests that acetylation of the hydroxyl group of quercetin significantly increased its intracellular absorption.

### 2.6. 4Ac-Q Bolsters the Metabolic Stability to Elongate Its Intracellular Persistence

To clarify the unknown metabolites in quercetin and 4Ac-Q-added HepG2 cells, we next investigated these metabolites by liquid chromatography–mass spectrometry (LC-MS) analysis based on HPLC-UV/Vis profiling.

**Figure 7:**
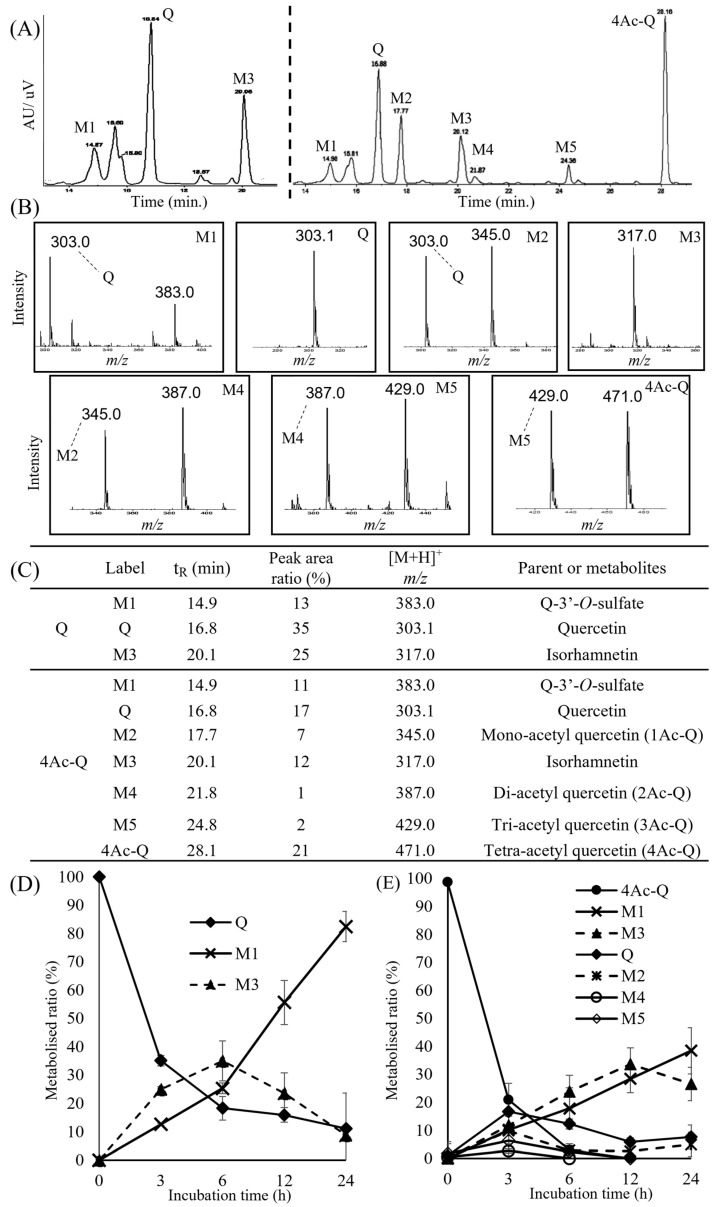
LC-MS analysis of HepG2 cell culture medium after treatment with quercetin(Q) and 4Ac-Q for the indicated times. (**A**) HPLC chromatograms of metabolites of quercetin (**left** side) and 4Ac-Q (**right** side). (**B**) Extracted ion chromatograms corresponding to different *m/z* values for the peaks of the HPLC chromatograms. (**C**) List of metabolites predicted based on the results of LC-MS analysis. Time-course changes in metabolites of quercetin (**D**) and 4Ac-Q (**E**) in HepG2 cells. Each graph was derived from the average of three independent measurements, respectively.

HPLC-UV/Vis profiling of quercetin detected two major metabolites (M1 and M3), excluding quercetin itself. Similarly, HPLC-UV/Vis profiling of 4Ac-Q detected five major metabolites (M1–M5), excluding both quercetin and 4Ac-Q. These HPLC profiling peaks were identified via electrospray ionization mass spectrometry (ESI-MS) analysis. In particular, the peaks corresponding to quercetin and 4Ac-Q were identified by co-elution with standard substances. The mass spectrum of quercetin metabolites showed that the [M+H]^+^ ions of M1 and its fragment ions were detected at *m/z* 383.0 and *m/z* 303.0, respectively, and the [M+H]^+^ ions of M3 were detected at m/z 317.0, suggesting that M1 and M3 may be quercetin-3′-sulfate and isorhamnetin, respectively (Figure 7B). The mass spectrum of 4Ac-Q metabolites showed that the [M+H]^+^ ions of M1, M2, M3, M4, and M5 were detected at *m/z* 383.0, 345.0, 317.0, 387.0, and 429.0, respectively. These data suggested that M1, M2, M3, M4, and M5 would be Q-3′-*O*-sulfate, Mono-acetyl quercetin (1Ac-Q), isorhamnetin, di-acetyl-quercetin (2Ac-Q), and tri-acetyl-quercetin (3Ac-Q), respectively (Figure 7C). Additionally, there were no detectable quercetin-glucuronides or glucuronide conjugates of 3′-methylquercetin from LC–MS data.

Furthermore, the time courses of the metabolite formation of quercetin and 4Ac-Q were shown in Figure 7D,E. The HepG2 cells were incubated with either quercetin or 4Ac-Q for 0, 3, 6, 12, or 24 h. The graph of quercetin metabolic rates showed that quercetin decreased in a time-dependent manner; conversely, metabolite M1 increased significantly over time. The quercetin metabolite M3 decreased after 6 h. On the other hand, 4Ac-Q decreased in a time-dependent manner and disappeared at 12 h. Quercetin, as one of the metabolites of 4Ac-Q, peaked at 3 h and decreased over time, and metabolite M1 increased in a time-dependent manner. However, compared to quercetin metabolism in Figure 7D, the percentage of metabolic conversion to M1 was found to be low. Interestingly, the metabolite M3 showed different patterns between quercetin and 4Ac-Q metabolism. M3 peaked at 6 h and then reduced during quercetin metabolism, while M3 peaked at 12 h and then reduced during 4Ac-Q metabolism. These data indicated that a portion of 4Ac-Q is transformed into quercetin, as well as several different acetylated quercetins during its metabolism. This partial deacetylation may have caused a delay in the metabolism of quercetin.

## 3. Discussion

Acylated polyphenols from natural plant sources are often modified in glycosidic sugar chains and have been reported to stabilize pigment and structure [22,23] and to enhance cytoprotective, antioxidant, and anti-inflammatory effects [24,25,26]. In contrast, there are few reports of acylation of aglycones in nature and few information on *O*-acylated flavonoids [10,12]. Thus, we synthesized 4Ac-Q via acetylation of its hydroxyl group. The structural confirmation was carried out via nuclear magnetic resonance (NMR) spectral analysis. It has been reported that a new chemical shift (δ) in the signal, indicative of the acetyl group around δ = 2.3 ppm, is a distinctive feature of 4Ac-Q resulting from the acetylation of hydroxyl groups at the 3,7,3′,4′-positions [18,27]. The four new signal peaks in our synthesized 4Ac-Q were observed to be attributed to the acetyl group, appearing at δ = 2.34 ppm and 2.37 ppm, which confirmed the synthesis was successful. The signal attributions and integration ratios aligned perfectly with previous reports [18,27], further substantiating the acetyl modification of the hydroxyl group.

In this study, 4Ac-Q exhibited significantly stronger inhibition of cell proliferation compared to quercetin. Our previous experiments with HL-60 cells revealed that the IC_50_ value of 4Ac-Q and quercetin was 19 μM and 58 μM, respectively, which resulted in a 3.05-fold higher inhibitory effect on cell proliferation for 4Ac-Q compared to quercetin. These data suggested that 4Ac-Q also exhibits higher inhibitory activity than quercetin, even in adherent cells. Moreover, quercetin with all hydroxyl groups acetylated also showed 2.59 times stronger inhibition of cell growth than quercetin in MCF-7 breast cancer cells [13]. Although the cell types and the number of acetyl modifications differ, acetylation may enhance the anticancer activity of quercetin. It is necessary for further evaluation of the effects of changes in the number and position of acetyl groups on physiological activity and cellular characteristics. On the other hand, Rubio et al. reported that quercetin 3-methyl ether tetraacetate induced apoptosis in HL-60 cells via the activation of caspases with cytochrome *c* release [28]. Thus, the activation pathways of the apoptosis-inducing proteins are possibly not determined by the acetylation site. In future studies, it is also required to investigate relative enzyme activity inhibition as part of quercetin acetylation’s anticancer efficacy. Quercetin is known to target various molecular kinase enzymes including Raf-1, MEK1 [29], ERK1/2, SEK1, JNK1/2, RSK2, and PI3K [30]. Silico analysis using a molecular docking model has reported that quercetin is bound to the same ATP-binding region as the specific PI3K inhibitor LY294002, which is considered a promising target for cancer therapy. Moreover, the slight differences in the mode of binding to the target protein can alter the inhibitory effect on PI3K [31]. It is interesting that a methylated derivative of quercetin effectively inhibited matrix metalloproteinase-1 (MMP-1), an enzyme involved in cancer invasion and metastasis, with a more potent effect than quercetin itself [32]. Methylated quercetin was reported to inhibit MMP-1 by binding near its active center, the metal ion, suggesting the position of the methyl group and the type of substituent may vary based on the specific protein. Therefore, future studies should examine the acetylation and other substituent modifications of hydroxyl groups, their positions and numbers, and the bioactivity of specific target proteins.

As an important aspect of this study, our data revealed that 4Ac-Q was taken up into the cell in larger amounts in a shorter time than parent quercetin and metabolized slowly (Figure 6 and Figure 7). The hydroxyl group of quercetin has been reported to be a target for glucuronidation and sulfate conjugation in phase II metabolism in the liver [33]. The hydroxyl groups of 4Ac-Q are replaced by the acetyl group, which may be prevented from these metabolic processes. Methylated flavonoids have been reported to prolong the metabolic stability [34]. In this study, more than 80% of quercetin was converted to sulfate conjugate in 24 h treatment in HepG2 cells (Figure 7D). On the other hand, only 40% of 4Ac-Q was converted to sulfate conjugate in 24 h treatment. Interestingly, 4Ac-Q itself disappeared after 12 h of incubation, while the monoacetylated quercetin was produced and remained in unmetabolized form after 24 h (Figure 7E). These data indicated that 4Ac-Q was more resistant to metabolism than quercetin.

Acetylation of (-)-epigallocatechin-3-*O*-gallate (EGCG) is reported to enhance a 30-fold cellular uptake and a 2.2-fold half-life in plasma elimination compared to EGCG [35]. Similarly, the conversion of the methyl group to an acetyl group at the C5 position of tangerine, a polymethoxyflavonoid, increased cellular uptake and prolonged its elimination half-life in mouse plasma [36]. Mono-acylated luteolin derivatives are considered to protect luteolin from metabolic activity [37]. These studies suggest that acetylation of the hydroxyl group of flavonoids may improve cellular uptake and avoid rapid metabolism, which is largely in agreement with the 4Ac-Q results in this study.

The increase in cellular uptake of quercetin acetyl derivatives was thought to be due in part to the change in polarity resulting from the substitution of the hydroxyl group with an acetyl group, thereby increasing bioaccessibility. Carrasco-Sandoval reported that the bioaccessibility of various phenolic compounds depended mostly on the chemical properties, especially their polarity [38]. Additionally, aglycones of flavonoids tend to form aggregates due to hydrophobic interactions among aromatic moieties and the hydrogen bonding of hydroxyl groups. This phenomenon is considered a key factor that reduces their solubility and restricts their bioavailability [39,40]. Based on our data combined with other reports, it can be inferred that the *O*-acetylation of quercetin may mitigate this aggregation by obstructing the formation of hydrogen bonds between hydroxyl groups and altering the compound’s polarity. Indeed, the crystal packing diagram of our single-crystal X-ray structure analysis of pentaacetyl quercetin shows that hydrogen bonding is inhibited by the presence of acetyl groups [41]. These observations align with the previous chemical and physicochemical findings [38], [39], and [40], supporting the hypothesis that acetylation plays a significant role in altering the behavior of phenolic compounds. On the other hand, deacetylation of 4Ac-Q in the metabolic process was thought to occur via hydrolysis by human-carboxylesterase 1 (hCE1) and human-carboxylesterase 2 (hCE2) present in HepG2 cells [42,43,44]. hCE2 especially prefers substrates with small acyl groups and large alcohol groups [45]; for example, it hydrolyzes benzoyl ester and the acetyl group of aspirin [46,47]. Thus, we considered that the deacetylation of 4Ac-Q in this study may also involve hCE2 reaction. It is necessary to confirm the active state of hCE2 in the metabolic process of 4Ac-Q in the next study.

In summary, our findings provide the first evidence that acetyl modification of quercetin not only substantially augments the intracellular absorption but also bolsters its metabolic stability, thereby prolonging its intracellular persistence. This implies that acetylation could serve as a strategic approach to enhance the bioactivity efficacy of quercetin and analogous flavonoids. Investigations on the structure–activity relationships of the acetylation of flavonoids remain a critical area for future exploration.

## 4. Materials and Methods

### 4.1. Chemicals and Antibodies

Quercetin was purchased from Sigma-Aldrich (St. Louis, MO, USA). Stock solution of sample was prepared in DMSO and diluted with complete culture media immediately before use. An equal volume of DMSO was added to controls. MTT and β-Actin antibody were purchased from Sigma-Aldrich LLC (St. Louis, MO, USA). The antibodies against caspase-3, caspase-9, PARP, cytochrome *c*, COX-4, and GAPDH were from Cell Signaling Technology (Danvers, MA, USA), whereas the antibodies against XIAP were from Abcam (Cambridge, MA, USA). SMAC/Diablo antibody was from Proteintech (Tokyo, Japan).

### 4.2. Synthesis of 4Ac-Q

4Ac-Q was prepared using the chemical synthesis method as described previously [18]. In brief, acetic anhydride (4 equiv.) was added to a quercetin solution (5 mmol) in dry pyridine at room temperature. After stirring the mixture for 10 min, it was quenched in ice-cold water. The resulting precipitate was collected by filtration and subsequently washed with ice-cold water. The compound was then recrystallized from methanol. The chemical structures of the acetylated quercetin were characterized by ^1^H-NMR. Spectra were recorded on a JEOL ECA-600 spectrometer (600 MHz for ^1^H, JEOL, Tokyo, Japan) using [D6]CDCl3 as the solvent and tetramethylsilane (TMS) as the internal standard. The purity was confirmed to be >97% by HPLC.

### 4.3. Cell Culture

Human hepatocellular carcinoma cell lines HepG2 were obtained from RIKEN Cell Bank (RCB1648) (Ibaraki, JP). The cells were cultured at 37 °C in a 5% CO_2_ atmosphere in Dulbecco’s-Modified Eagle Medium (DMEM) containing 10% fetal bovine serum and 1% of penicillin–streptomycin glutamine for 24 h and then treated by samples in indicated times and doses.

### 4.4. Cell Viability Assay

Cell viability was determined by an MTT assay, as described previously [48]. In brief, HepG2 (9.35 × 10^4^/well) cells were plated into each well of 12-well plates. The cells were treated with 40 or 80 μM of samples for 48 h. MTT solution was then added to each well and incubated for another 4 h. The resulting MTT–formazan product was dissolved by the addition of DMSO. The amount of formazan was determined by measuring the absorbance at 550 nm with Multiskan TM FC (Thermo Scientific^TM^, Waltham, MA, USA). The cell viability was expressed as the optical density ratio of the treatment to control.

### 4.5. Apoptosis Detection by Annexin V-FITC/Propidium Iodide Flow Cytometry

Apoptosis induction was quantitatively assessed via flow cytometry using the FITC Annexin V Apoptosis Detection Kit I (BD Biosciences, San Diego, CA, USA) according to the manufacturer’s manual [48]. In brief, HepG2 (2.4 × 10^5^/Well) was plated into each well of 6-well plates. After treatment with 80 μM of samples for 24, 48, or 72 h, HepG2 cells were suspended in 100 μL of binding buffer and then incubated with FITC Annexin V and PI staining solution for 15 min. The cells were analyzed at FL1 (530 nm) and FL3 (630 nm) with the flow cytometry (CyFlow^®^, Sysmex Partec GmbH, Görlitz, Germany).

### 4.6. Protein Array Analysis of Apoptosis-Related Proteins

Proteome profiling of apoptosis-related protein was assessed with proteome profiler human apoptosis array kit (R&D Systems, Milpitas, CA, USA) according to the manufacturer’s manual. Briefly, HepG2 cells were treated with DMSO control, 80 µM of quercetin or 4Ac-Q, then cultured for 24 h, and each cell lysate was prepared in the array kit lysis buffer. Cell lysates are diluted and incubated overnight at 4 °C with nitrocellulose membranes containing various duplicates of 35 different apoptotic antibodies to detect proteins. The membranes were washed to remove unbound proteins, followed by incubation with a cocktail of biotinylated detection antibodies. Then, the levels of apoptosis-related proteins were assessed using an HRP-conjugated antibody followed via chemiluminescence, and each array membrane was scanned using LumiVision Analyzer140 .exe (TAITEC, Saitama, Japan).

### 4.7. Western Blot Analysis

HepG2 cells were treated with DMSO (control), Quercetin, or 4Ac-Q for defined times and then collected by centrifugation at 1700× *g* for 10 min. After the cells were lysed as described previously [48], equal amounts of lysate protein were run on sodium dodecyl sulfate polyacrylamide gel electrophoresis (SDS-PAGE) and electrophoretically transferred onto polyvinylidene difluoride (PVDF) membrane (GE Healthcare UK, Amersham, England). Immunoblotting was performed following our previous method [48]. Bound antibodies were detected using the enhanced chemiluminescence (ECL) system, and relative amounts of proteins associated with specific antibodies were quantified using LumiVision Analyzer (TAITEC, Saitama, Japan).

### 4.8. Subcellular Fractionation for Cytochrome c Detection

Mitochondria were prepared as described in our previous study [48] using the mitochondria/cytosol fractionation kit (BioVision, Milpitas, CA, USA). The harvested cells were suspended in a cytosol extraction buffer. After incubation on ice for 10 min, cells were homogenized and centrifuged at 700× *g* for 10 min. The supernatant was further centrifuged at 10,000× *g* for 30 min., and the cytosol and mitochondrial fractions were isolated. The supernatant was designated as the cytosol fraction. The pellet was dissolved in mitochondria extraction buffer and used as the mitochondrial fraction.

### 4.9. Determination of Mitochondrial Membrane Potential (ΔΨm)

Mitochondrial membrane potential in HepG2 cells was determined by flow cytometry as described previously [49] using the JC-1 MitoMP Detection Kit (Dojindo Molecular Technologies, Kumamoto, Japan). HepG2 cells (2.4 × 10^5/^well) were seeded in a 6-well plate and treated with DMSO, 80 μM quercetin, or 4Ac-Q for 0.5 h. Afterward, the cells were incubated for 30 min with JC-1 (2 μg/mL) in the dark. After incubation, the cells were washed twice with hanks’ balanced salt solution (HBSS), suspended in a total volume of 200 μL of imaging buffer solution, and analyzed using a flow cytometer.

### 4.10. Determination of ROS Production

ROS production was measured via flow cytometry using MitoSOX^TM^ Red mitochondrial superoxide indicator (Invitrogen-Life Technologies, Carlsbad, CA, USA) as described previously [49]. For flow cytometric analysis, HepG2 (2.4 × 10^5^/well) cells were plated into each well of 6-well plates. After treatment with 80 μM of samples for 30 min, HepG2 cells were incubated with 50 μM MitoSOX Red for 10 min. The cells were collected by centrifugation at 1700× *g* for 10 min and washed with phosphate-buffered saline (PBS). The fluorescence was detected using the flow cytometer.

### 4.11. Uptake of Quercetin and Acylated Quercetin into HepG2 Cells

The uptake of quercetin and 4Ac-Q into HepG2 cells was measured by the method of Wong et al. [50]. The cells were culturedto 80% confluent, and the medium was changed every 2 days. Uptake experiments were performed by adding 80 µM quercetin or 4Ac-Q to a serum-free medium. After 3 h of sample treatment, the supernatant was removed, and the pellet was washed two times with ice-cold PBS containing 0.2% bovine serum albumin (BSA) and one time with ice-cold PBS without BSA. The cells were collected with 50% methanol and stored at −80 °C for at least 24 h. Extraction was performed by sonication for 10 min, followed by the addition of ice-cold acetone to twice the volume of 50% methanol. The samples were placed in a −20 °C freezer for 1h and centrifuged at 17,000× *g* for 5 min. The supernatant was collected and evaporated for dryness in vacuo at 30 °C and stored at −20 °C until HPLC analysis.

### 4.12. HPLC Analysis

Cellular uptake of samples was analyzed via HPLC unit (LC-2000Plus series; JASCO Corporation, Tokyo, Japan) using COSMOSIL(R) πNAP packed column 4.6 mm I.D. × 250 mm (Nacalai Tesque, Kyoto, Japan). The solvent system was a mixture of water (A) and acetonitrile (B) with a flow rate of 1.0 mL/min and a gradient of 100% B for 0–25 min and 20% B for 25–30 min, starting with an A/B ratio of 80:20, returning to the initial condition for 10 min. A photodiode array (PDA) detector was used for scanning, accumulating spectral data for all peaks in the absorption wavelength range 200–700 nm and recording chromatograms at 280, 320, and 370 nm, respectively.

### 4.13. Preparation of Metabolites of Quercetin and Acylated Quercetin in HepG2 Cells

Metabolites in HepG2 cells were measured based on the method of O’Leary et al. [51] with a few modifications. The cells were seeded in 58 cm^2^ dishes at a density of around 1.12 × 10^5^ cells/cm^2^ and allowed to adhere overnight. Cells were cultured to 80% confluent, and the medium was changed every 2 days. On the day of the experiment, cells were washed twice with PBS, and a fresh medium was added. The cells were further incubated with 80 μM of quercetin or 4Ac-Q for 0, 3, 6, 12, or 24 h, respectively. At each time, the supernatant medium was collected and immediately cooled on ice. The medium was processed for HPLC analysis as follows: cold methanol containing 1 mM ascorbic acid was added 1.5 times as much as the collected medium. Samples were centrifuged at 13,500× *g* for 10 min at 4 °C. The pellet was dried by rotary evaporation and resuspended in 1 mL of 50% acetonitrile. Samples were filtered through a 0.22 µm filter prior to analysis by HPLC and LC-MS.

### 4.14. Liquid Chromatography–Mass Spectrometry (LC-MS) Characterization of Metabolites

For LC/MS analysis, a QTRAP^®^ liquid chromatography-electrospray ionization-tandem mass spectrometry (LC-ESI-MS/MS) 3200 system (1.7 with HotFix 2, AB Sciex Pte. Ltd., Framingham, MA, USA) was connected to the LC20 HPLC instrument (Shimadzu Corporation, Kyoto, Japan) via an ESI interface. The analytical column was a phase C18 (octadecyl) (TSKgel ODS-100V, L × I.D. 150 mm × 3.0 mm, 3 μm particle size; Tosoh Corporation, Tokyo, Japan) equipped with a UV detector (280 nm) at a flow rate of 0.4 mL/min and with an injection volume of 10 μL. The mobile phase was solvent A (99.9% water + 0.1% formic acid) and solvent B (99.9% acetonitrile + 0.1% formic acid), starting with 10% solvent B at 0 min and gradient to 100% solvent B for 45 min. The mass spectrometer was monitored in the positive mode. The optimized detection parameters were as follows: scan type, electrospray ionization (ESI), ionization mode: positive mode, curtain gas: 20 psi, collision gas: High, ion spray voltage: 5.5 kV, temperature: 600 °C, ion source gas1: 50 psi, ion source gas2: 80 psi, and interface heater: ON.

### 4.15. Statistical Analysis

Statistical analysis of data was determined by one-way analysis of variance (ANOVA) followed by Tukey’s multiple comparison test, using SPSS v26 and GraphPad Prism 9 software. Data are expressed as the mean ± standard deviation (SD). All experiments were conducted in biological triplicates (n = 3) with at least three individual replicates, and uptake and metabolism experiments generated at least three biological replicates. The significance level was set as p-values less than 0.05.

## 5. Conclusions

Hydroxyl group acetylation of quercetin enhances its intracellular absorption and metabolic stability, which strengthen the anticancer activity, including cell growth inhibition and apoptosis induction of cancer cells. This innovative approach will provide a novel strategy against cancer cell growth, not only for quercetin but also for related flavonoids.

## Data Availability

The data used to support the findings of this study are available from the corresponding author upon request.
